# Topical versus systemic tranexamic acid after total knee and hip arthroplasty: A meta-analysis of randomized controlled trials

**DOI:** 10.1097/MD.0000000000004656

**Published:** 2016-10-14

**Authors:** Yongcai Chen, Zhuo Chen, Shuo Cui, Zhiyang Li, Zhengjiang Yuan

**Affiliations:** a Department of Microsurgery, The First Affiliated Hospital and College of Clinical Medicine of Henan University of Science and Technology; b Medical College of Henan University of Science and Technology, Luoyang, Henan, China.

**Keywords:** hip arthroplasty, intravenous, knee arthroplasty, topical, tranexamic acid

## Abstract

**Background:**

Tranexamic acid (TXA) is an antifibrinolytic drug widely used to reduce blood loss during joint replacements, including total knee arthroplasty (TKA) and total hip arthroplasty (THA). However, there is no final consensus regarding the composition of an optimal administration of TXA regime between topical and systemic (intravenous). The purpose of our study was to compare the efficacy of topical and intravenous (IV) regimen of TXA during TKA and THA.

**Methods:**

Five relevant electronic online databases, PubMed, EMBASE, the Cochrane Central Register of Controlled Trials, Web of Science and Chinese Biomedical Database were systematically searched in November 2015. Randomized controlled trials (RCTs) that compared topical with intravenous TXA in patients with TKA or THA were included. The search terms included “topical,” “intravenous,” “tranexamic acid,” “knee arthroplasty” and “hip arthroplasty.” Two reviewers independently extracted data and assessed the risk of bias and study quality. Data were analyzed with Review Manager 5.3 software. Grades of Recommendation Assessment, Development and Evaluation (GRADE) were used to assess the quality of evidence.

**Results:**

Sixteen RCTs with 1250 patients undergoing TKA and 4 RCTs involving 550 patients undergoing THA were included. There were no significant differences in total blood loss (mean difference [MD]_TKA_ = −28.72 mL, 95% confidence interval [CI] −195.97 to 138.54 mL, *P* = 0.74; MD_THA_ = 14.03 mL, 95% CI −35.53 to 63.59 mL; *P* = 0.78), total drain out (MD_TKA_ = −3.09 mL, 95% CI −39.05 to 32.88 mL; *P* = 0.87; MD_THA_ −31.00 mL, 95% CI −66.56 to 4.66 mL; *P* = 0.09), and transfusion rates (OR_TKA_ = 0.90, 95% CI 0.58–1.40, *P* = 0.64; OR_THA_ = 1.19, 95% CI 0.67–2.09; *P* = 0.63) between topical and intravenous (IV) TXA.

**Conclusions:**

The current evidence suggested that topical TXA was equally effective and safe compared with intravenous TXA in reducing blood loss and transfusion rate following TKA or THA. We recommended that either topically or systemically could be used in TKA and THA to decrease perioperative blood loss.

## 1 Introduction

It is reported that >700,000 joint replacements are performed in the United States each year.^[1]^ Total knee arthroplasty (TKA) and total hip arthroplasty (THA) are among the most successful procedures for patients with painful degenerative knee or hip diseases. However, joint replacements are often associated with substantial amounts of blood loss, for example, blood loss in TKA ranges from 500 to 1500 mL and in THA, ranges from 1188 to 1651 mL.^[2,3]^ To counter this blood loss, an average of approximately 2 units of allogeneic blood transfusions are often required to optimize the postoperative decrease in hemoglobin (Hb) and hematocrit concentrations.^[4]^ Perioperative blood loss and allogenic transfusions can potentially result in substantial cost increases and significant complications, such as postoperative infection, delayed physical recovery, longer hospital stays, and increased mortality.^[5]^

Some strategies have been employed to reduce blood loss and transfusion requirements in joint replacements, including preoperative autologous donation, cell salvage, controlled hypotension, regional anesthesia, and the use of erythropoietin and antifibrinolytics.^[2]^ As an antifibrinolytic agent, tranexamic acid (TXA), that is a synthetic analog of the amino acid lysine, has been widely used in joint replacements.^[6]^ Meta-analyses have found the efficacy of TXA for reducing blood loss and decreasing transfusion rates in both TKA^[7,8]^ and THA.^[9]^

Three routes of TXA administration have been reported in the literatures, that is intravenous (IV),^[10]^ topical,^[11]^ and oral.^[12]^ Compared to IV administration, topical TXA is usually given as a topical wash or into the knee joint after wound closure via the drain; the benefits of topical TXA include ease of administration, ability to achieve maximum concentration at the bleeding site, and minimal systemic absorption.^[11,13]^ Recently, some randomized controlled trials^[14–33]^ have compared topical TXA with IV TXA in joint replacements. However, the conclusions among studies are still controversial. Some studies^[15,18,19,23]^ found no differences between topical and IV TXA regarding blood loss, whereas others studies supported the topical^[14,17]^ or IV TXA^[16]^ for its less blood loss.

Therefore, we conducted a meta-analysis of randomized controlled trials (RCTs) to compare the topical with IV TXA in patients undergoing primary TKA or THA. The hypothesis of this meta-analysis was that topical TXA is as good as systemic TXA for reducing blood loss and transfusion rates?

## 2 Methods

Our meta-analysis was conducted in accordance with the guidelines described in the Cochrane handbook for systematic review and meta-analysis of interventions^[34]^ and Preferred reporting items for systematic reviews and meta-analyses (PRISMA) statement.^[35]^

### 2.1 Search strategy

Five bibliographic databases including PubMed, Embase, the Cochrane Library, Web of Science and Chinese Biomedical Database were searched to identify RCTs comparing topical with IV TXA for patients with TKA and THA. The literature search was conducted in November 2015 without restriction to regions, publication types, or languages. A systematic search strategy was developed and the search terms were: tranexamic acid, topical, IV, knee, hip, arthroplasty and replacement. A manual search of the reference lists of all included papers and relevant review was also conducted for further eligible studies.

### 2.2 Inclusion criteria

To be eligible for inclusion, studies needed to fulfill the following criteria: randomized controlled trials that compared topical with IV TXA in patients who underwent TKA or THA. Two reviewers screened and assessed the eligibility of all studies on the basis of these criteria. Disagreement was discussed with a third investigator.

The primary outcome we evaluated was transfusion rates (the numbers of patients who underwent allogenic blood transfusion). The secondary outcomes included means and standard deviations of change in haemoglobin concentration from before surgery to different postoperative time, total blood loss, darin output, length of hospital stay, and complications (deep venous thrombosis (DVT) and Pulmonary Embolism (PE)).

### 2.3 Data extraction and quality assessment

Two independent reviewers used a standardized data extraction tool to abstract information about patient populations, age, settings, study designs, interventions and follow-ups. Disagreements about extractions were resolved through discussion by another investigator.

We assessed the quality of included RCTs by using predefined criteria from the Cochrane Risk of Bias tool^[34]^ that included adequacy of randomization, allocation concealment, incomplete outcome data (attrition bias), selective reporting (reporting bias), and other bias.

We assessed strength of evidence for each major outcome according to the Grading of Recommendations Assessment, Development, and Evaluation (GRADE) criteria.^[36]^ Both investigators made judgments on risk for bias, precision, consistency, directness, and likelihood of publication bias.

### 2.4 Statistical analysis

The odds ratio (OR) and its corresponding 95% confidence interval (CI) were calculated for dichotomous data, and the continuous data were summarized using the mean difference (MD) and its corresponding 95% CI. We used the *I*^2^ statistic to assess statistical heterogeneity: a value of *I*^2^ >50% represented heterogeneity. Where heterogeneity existed, random-effects models were used; otherwise, fixed-effect models were acceptable. Statistical analyses were performed using Review Manager 5.3 software (RevMan 5.3, The Cochrane Collaboration, Oxford, UK). *P* value was <0.05 being considered as statistically significant. This is a meta-analysis of literatures, so ethical approval was not necessary for our research.

## 3 Results

### 3.1 Search results and study characteristics

**Figure 1:**
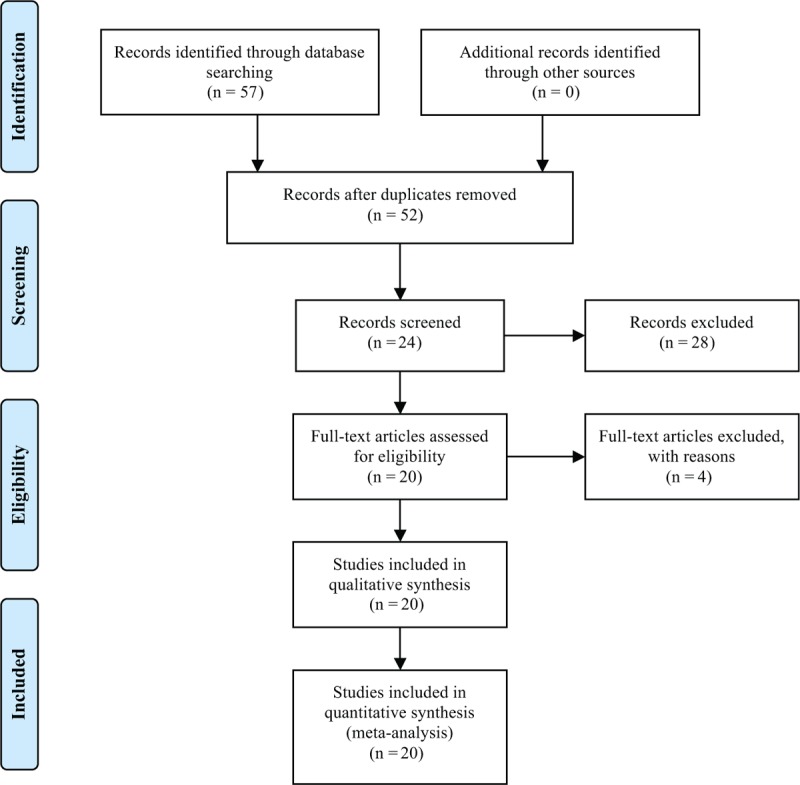
PRISMA Flow Diagram.

The electronic search originally identified 57 citations. Of these, 24 were deemed potentially eligible by reading title and abstracts. After screening and full-text articles, eventually, 20 studies^[14–33]^ involving 1800 patients met the inclusion criteria (Fig. 1).

Of these, 16 RCTs^[14–18,20–26,28,29,32,33]^ with a total of 1250 patients investigated TXA in patients undergoing TKA and 4 (550 patients)^[19,27,30,31]^ in THA. These studies involved a total of 899 patients in the topical TXA group and 901 patients in the IV TXA group. All trials were published between 2012 and 2015. Participant numbers ranged from 40 to 203. The dose of topical TXA ranged from 1 to 3 g and the IV-TXA ranged from 10 to 30 mg/kg. The baseline parameters of included studies were comparable between the topical TXA and IV TXA group, including age, preoperative haemoglobin, body mass index, and operative time (Table 1).

**Table 1:**
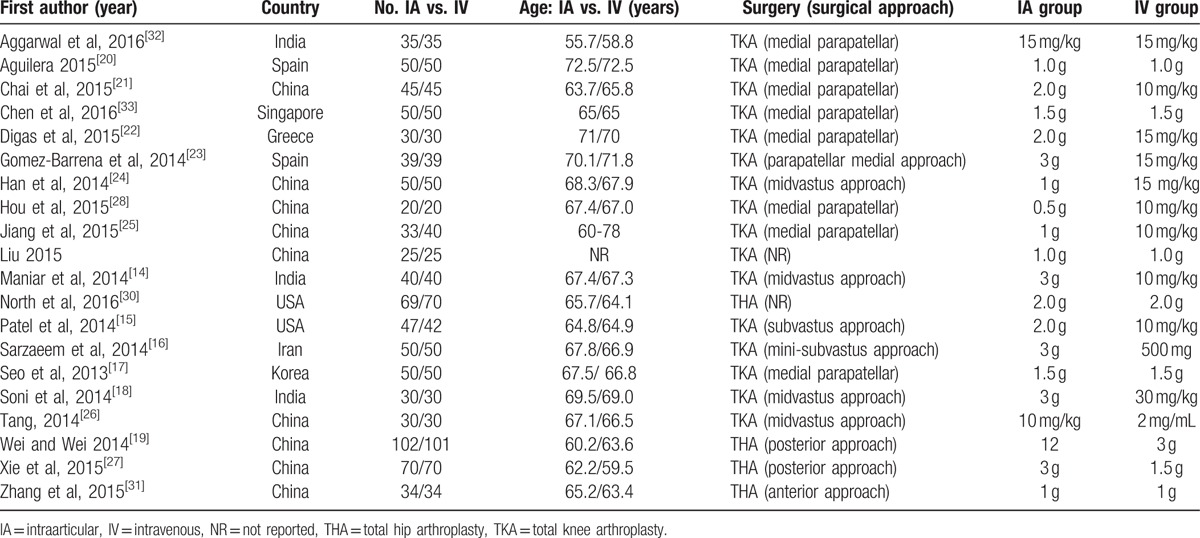
Characteristics of included studies.

According to the Cochrane Collaboration guidelines, all the included studies were randomized, but only 10 studies^[15–21,31–33]^ provided the detailed method of random generation. Only 7 studies^[14,19,22,23,27,32,33]^ referred to information with regard to allocation concealment. There was an unclear bias in 11 studies^[17,18,20,21,24–26,28,29,31]^ in the blinding measurement. No other sources of bias were detected in these studies (Table 2).

**Table 2:**
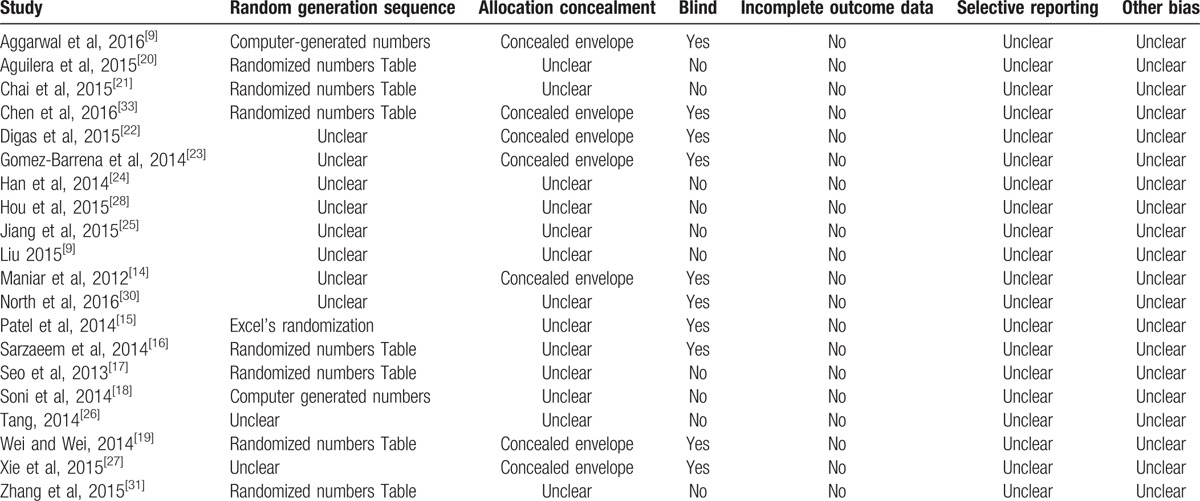
Risk of bias in included studies.

### 3.2 Total knee arthroplasty

Totally, 16 RCTs^[14–18,20–26,28,29,32,33]^ with a total of 1250 patients investigated TXA in patients undergoing TKA.

#### 3.2.1 Primary outcomes

##### 3.2.1.1 Transfusion rates

Twelve studies^[14–18,20–24,33]^ involving 1026 patients provided usable data of transfusion rates. Meta-analysis showed that there was no statistical difference in transfusion rates between topical TXA group and the IV TXA group (OR = 0.90, 95% CI 0.58–1.40; *P* = 0.64). No significant heterogeneity was observed (*I*^2^ = 30%).

#### 3.2.2 Secondary outcomes

##### 3.2.2.1 Blood loss

All included studies reported the result of total blood loss, hidden blood loss, and total drain output. The results of meta-analysis demonstrated that there were no significantly statistical differences in total blood loss (MD = −28.72 mL, 95% CI −195.97 to 138.54 mL, *P* = 0.74), total drain output (MD = −3.09 mL, 95% CI −39.05 to 32.88 mL, *P* = 0.87), and hidden blood loss (MD = −43.45 mL, 95% CI −325.25 to 238.26 mL, *P* = 0.76) between topical TXA group and IV TXA group.

##### 3.2.2.2 Haemoglobin (Hb) drop

This outcome was described in 11 trials^[15–18,20–24,26,29,32,33]^ (495 patients in the topical TXA group and 491 patients in the IV TXA group). No significant differences in the drop of Hb were observed at day 1 (MD = 0.18, 95% CI −0.09 to 0.46, *P* = 0.19) and day 2 (MD = −0.59, 95% CI −1.40 to 0.23, *P* = 0.16) between Topical TXA and IV TXA group.

##### 3.2.2.3 Length of hospital stay

Three trials^[20,23,32]^ with 248 patients were available for the data of length of hospital stay. Meta-analysis showed that no significant difference was found in the length of hospital stay (MD = −0.77, 95% CI −1.65 to 0.10, *P* = 0.08) between the 2 groups. Significant heterogeneity was observed (*I*^2^ = 73%) among studies.

##### 3.2.2.4 Complications

All included trials provided usable data on the incidence of complications, including infection, deep vein thrombosis (DVT), or pulmonary embolism. Meta-analysis demonstrated that there was no significant difference in the incidence of infection (OR = 1.00, 95% CI 0.14–7.24, *P* = 1.00), DVT (OR = 1.10, 95% CI 0.45–2.68, *P* = 0.83) between the 2 groups. No significant heterogeneity was observed in all outcomes of infection and DVT (*I*^2^ = 0%).

### 3.3 Total hip arthroplasty

A total of 4 RCTs^[19,27,30,31]^ with 550 patients investigated the result of TXA in THA.

#### 3.3.1 Primary outcomes

##### 3.3.1.1 Transfusion rates

Four studies^[19,27,30,31]^ involving 550 patients provided usable data of transfusion rates. Meta-analysis showed that there was no statistical difference in transfusion rates between topical TXA group and the IV TXA group (OR = 1.19, 95% CI 0.67–2.09, *P* = 0.63). No significant heterogeneity was observed (*I*^2^ = 0%).

#### 3.3.2 Secondary outcomes

##### 3.3.2.1 Blood loss

All included studies reported the result of total blood loss, hidden blood loss, and total drain output. Meta-analysis demonstrated that there were no significantly statistical differences in total blood loss (MD = 38.66 mL, 95% CI −38.97 to 116.30 mL, *P* = 0.33), total drain output (MD = −31.00 mL, 95% CI −66.56 to 4.66 mL, *P* = 0.09), and hidden blood loss (MD = −5.84 mL, 95% CI −38.44 to 26.75 mL, *P* = 0.73) between topical TXA and IV TXA group.

##### 3.3.2.2 Hb drop

This outcome was described in 3 trials with a total of 482 patients. No significant differences in the drop of Hb at day 1 (MD = 0.30, 95% CI −0.08 to 0.68, *P* = 0.12) between Topical TXA and IV TXA group were observed.

##### 3.3.2.3 Length of hospital stay

Two trials^[19,27]^ with 343 patients were available for the data of length of hospital stay. Meta-analysis showed that no significant difference was found in the length of hospital stay (MD = −0.05, 95% CI −0.32 to 0.42, *P* = 0.80) between the 2 groups. No heterogeneity was observed (*I*^2^ = 0%) among studies.

##### 3.3.2.4 Complications

All included trials provided usable data on the incidence of complications, including infection, DVT, or pulmonary embolism. Meta-analysis demonstrated that there was no significant difference in the incidence of infection (OR = 1.52, 95% CI 0.25–9.49, *P* = 0.65), DVT (OR = 0.23, 95% CI 0.05–1.10, *P* = 0.07), and pulmonary embolism (OR = 0.33, 95% CI 0.01–8.32, *P* = 0.50) between the 2 groups. No significant heterogeneity was observed in all outcomes of infection, DVT, or pulmonary embolism (*I*^2^ = 0%).

### 3.4 Level of evidence of outcomes

According to the GRADE approach, we assessed the evidence level of main outcomes (Table 3). The evidence level of transfusion rates and DVT in patients with TKA or THA was high, and the remaining outcomes were moderate. The downgraded factors of moderate evidence were for inconsistency (high heterogeneity).

**Table 3:**
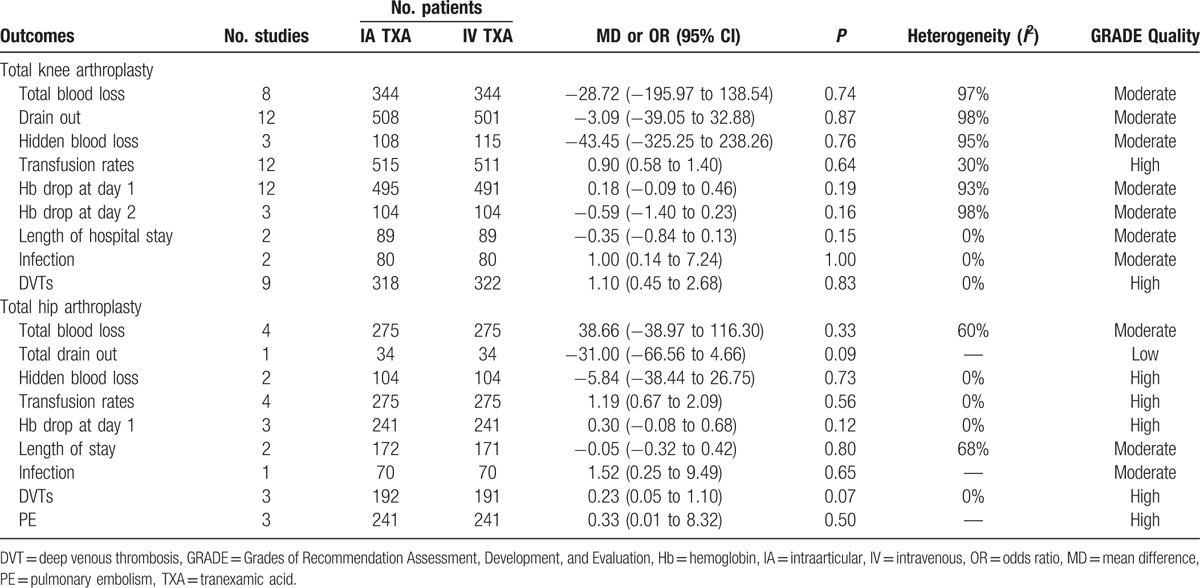
The outcomes of meta-analysis.

### 3.5 Publication bias

**Figure 2:**
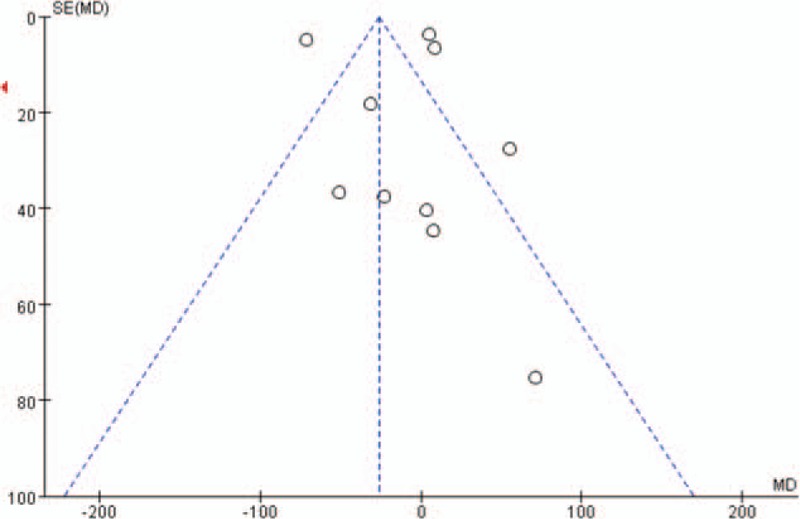
Funnel plot of the outcome total drain out demonstrates minimal publication bias.

**Figure 3:**
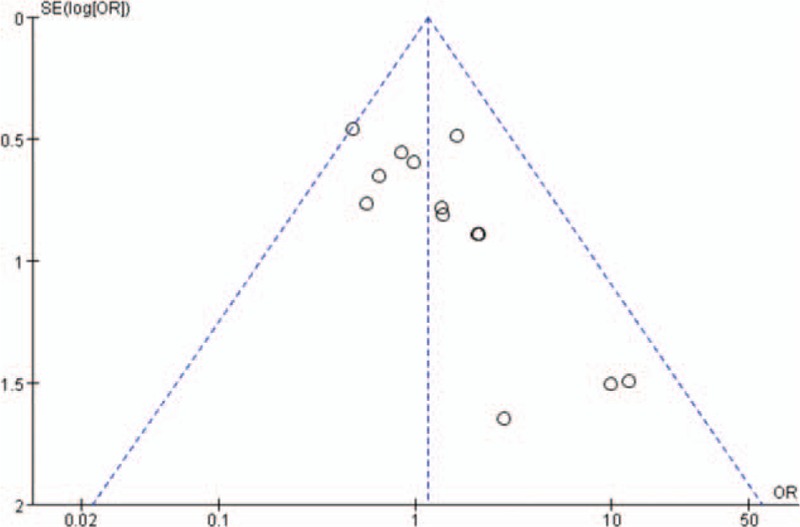
Funnel plot of the outcome transfusion rates demonstrates minimal publication bias.

**Figure 4:**
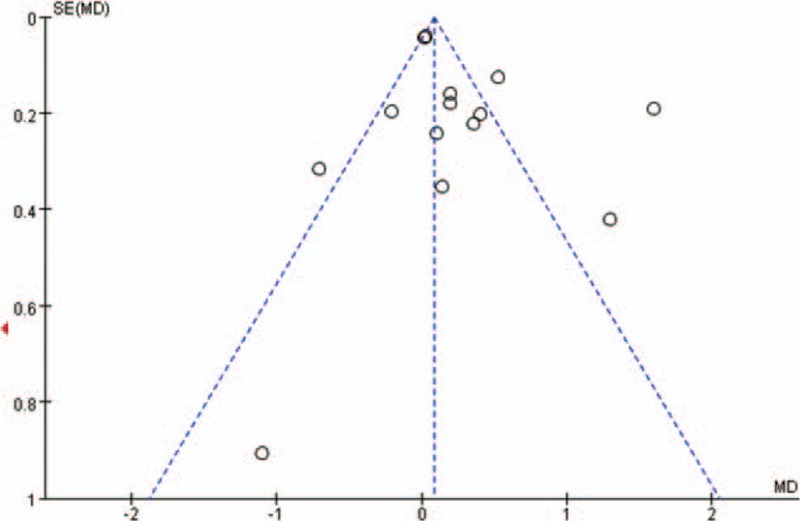
Funnel plot of the outcome Hb drop at day 1 demonstrates minimal publication bias.

Figures 2–4 represented funnel plots examining for potential publication bias among studies involving patients undergoing TKA or THA. Figure 2 reported the MD of drain out as a measure of the treatment effect (TXA). Figure 3 reported the logs OR of the transfusions rates as a measure of the treatment effect. Figure 4 reported the MD of the drop of Hb level as a measure of the treatment effect. All 3 Figures demonstrated only minimal asymmetry and a few outliers, indicating mild publication bias.

## 4 Discussion

### 4.1 Summary of findings

The most important findings of our meta-analysis were that there were no significant differences in transfusion rates, total blood loss, drain output, the drop of Hb level at day 1, and the incidence of infection and DVT between topical and IV TXA for patients with TKA or THA.

TXA is a synthetic antifibrinolytic agent and can competitively inhibit fibrinolysis by reversibly blocking the lysine-binding sites of plasminogen, thereby displacing plasminogen from the fibrin surface.^[37]^ The trauma of surgery promotes the release of tissue plasminogen activator and the activation of fibrinolysis, so TXA can block the activation process (plasminogen to plasmin) in an earlier stage and thus reduce blood loss postoperatively.^[10]^ Various methods of administration of TXA have been used, including oral, intraarticular injections, and IV.^[10,38,39]^ However, there remains no consensus regarding the optimal regimen for tranexamic acid administration.

When TXA is administered intravenously, it is widely distributed through the extracellular and intracellular compartments. The time required to reach maximum plasma levels of TXA is reportedly 5 to 15 minutes after IV injection.^[10]^ However, in some medical conditions, including renal insufficiency, history of previous DVT, and cerebrovascular and cardiac disease, the use of IV TXA was precluded. For this limitation in the use of IV TXA, some authors sought to support the use of topical TXA. In theory, patients at risk with the use of IV TXA may tolerate this medication topically at the operative site without increased risks of systemic adverse events.^[15]^ Topical injection at the surgical site provides a direct and straightforward means of application before tourniquet release. In addition, intraarticular TXA injection has the advantage of inducing partial microvascular hemostasis by stopping fibrin clot dissolution in the affected area. When the concentration of the TXA in synovial fluid reaches to the concentration of serum, it will diffuse into the synovial fluid and synovial membranes, and the biological half-life of TXA in the joint fluid is about 3 hours.^[38]^ Systematic reviews and meta-analyses have found the effectiveness and safety of IV TXA, and strongly recommend its use in joint replacements.^[7,9]^

In recent years, topical TXA has been proposed as an alternative and is applied predominantly to the TKA and THA.^[40]^ The potential advantages of topical application of tranexamic acid are directly targeting of the site of bleeding and preventing of systemic side effects.^[11]^ Alshryda et al^[8]^ conducted a meta-analysis of 14 RCTs to investigate the effect of topical TXA in joint replacements and found that topical TXA reduced the rate of blood transfusion in both TKA and THA without increasing the rates of DVTs. Owing to the lack of studies that directly compared IV TXA administration to topical one, Alshryda et al performed an indirect comparison of placebo-controlled trials of topical and IV TXA and demonstrated that topical administration was superior to the IV administration. However, the results from indirect comparison may reduce power of randomization and may systematically overestimate the effects of treatments.^[41]^ Therefore, we undertook this meta-analysis to directly compare topical TXA with IV TXA.

The primary outcome in our study is transfusion rates. Soni et al^[18]^ randomized 60 patients into topical TXA (30 patients) and IV TXA (30 patients) and found that topical TXA is equally effective as IV regimen in reducing blood loss during TKA. Patel et al^[15]^ compared efficacies and safety profiles of topical versus IV TXA in patients who underwent TKA and reported that topical TXA had a similar efficacy to IV TXA in reducing perioperative blood loss following primary TKA. Besides, Wei et al^[19]^ conducted an RCT to determine the results of topical TXA compared with IV TXA following THA. They concluded that topical use of TXA was equally effective and safe compared with IV TXA in reducing blood loss and transfusion rate in patients with THA without substantial complications. Consistent with previous studies, our meta-analysis revealed that there were no significant differences in blood loss and transfusion rates between the 2 groups.

Another important issue is the thromboembolic complications. Previous meta-analyses^[7–9]^ have confirmed that TXA did not increase the incidence of DVTs than placebo. In addition, topical applications of TXA were considered to cause infection. In our study, all included studies reported infection, DVTs, or pulmonary embolism complication data. Totally, 10 of 510 studies in the topical TXA group and 15 of 512 studies in the IV TXA had clinical suspicion of DVT, which did not reach statistical difference. The current evidence from trials did not find significant difference in the risk of infection and DVT between the 2 administration groups.

### 4.2 Strengths and limitations of this study

Three previous systematic reviews and meta analyses^[8,42,43]^ have reported on the same subject. Alshryda et al^[8]^ performed an indirect comparison of placebo-topical TXA trials to placebo-IV TXA trials. There was no published RCT that compared the 2 routes head to head. This has significantly limited the value of such comparison undermining their recommendation. The other 2 reviews^[42,43]^ included fewer studies: single study in the former and 5 studies in the latter. Our study has overcome the above limitations by including substantially larger number of studies that directly compared the 2 routes of administration. To the best of our knowledge, this is the largest systematic review and meta-analysis that has addressed this topic. Moreover, in various stages of conducting and reporting the review, we have strictly adhered to the highest standard recommended by Cochrane library, PRISMA guideline, and GRADE systems to ensure a high-quality review and meta-analysis.

Several limitations should be considered in this study. First, despite only RCTs were identified, some different factors may influence the results, such as surgical approach, blood management protocols, and VTE prophylaxis protocols. In addition, patients with high-risk factors were excluded, such as patients with cardiovascular disease, previous VTE events, and renal dysfunction, which means that the results of the analysis could not be extrapolated. Second, there were insufficient data to support the analysis of functional outcome scores or quality of life outcome measures. Third, the safety profile of both topical and IV TXA should be noted. Although there is no significant difference between the 2 groups, none of the included studies were powered to detect such a difference and even with the pooled (larger number) the findings remain underpowered to establish safety. Last, no cost benefit analysis was done for this study. Future studies should be designed to investigate not only the efficacy but also the cost benefit analysis between the 2 administrations.

## 5 Conclusions

The current evidence suggested that topical TXA was equally effective and safe compared with IV TXA in reducing blood loss and transfusion rate following TKA or THA. We recommended that TXA either topically or systemically could be used in TKA and THA to decrease perioperative blood loss.
